# Bioavailable constituents/metabolites of pomegranate (*Punica granatum *L) preferentially inhibit COX2 activity *ex vivo *and IL-1beta-induced PGE_2 _production in human chondrocytes *in vitro*

**DOI:** 10.1186/1476-9255-5-9

**Published:** 2008-06-13

**Authors:** Meenakshi Shukla, Kalpana Gupta, Zafar Rasheed, Khursheed A Khan, Tariq M Haqqi

**Affiliations:** 1 Division of Rheumatic Diseases, Department of Medicine, Case Western Reserve University, 10900 Euclid Avenue, Cleveland, OH 44106, USA; 2 Department of Kulliyat, Faculty of Unani Medicine, Aligarh Muslim University, Aligarh 202 002, India; 3 Department of Pathology, Microbiology & Immunology, School of Medicine, University of South Carolina, 6439 Garners Ferry Road, Columbia, SC 29209, USA

## Abstract

Several recent studies have documented that supplementation with pomegranate fruit extract inhibits inflammatory symptoms *in vivo*. However, the molecular basis of the observed effects has not been fully revealed. Although previous studies have documented the inhibition of nitric oxide and cyclooxygenase (COX) activity *in vitro *by plant and fruit extracts added directly into the culture medium but whether concentrations of bioactive compounds sufficient enough to exert such inhibitory effects *in vivo *can be achieved through oral consumption has not been reported. In the present study we determined the effect of rabbit plasma obtained after ingestion of a polyphenol rich extract of pomegranate fruit (PFE) on COX enzyme activity *ex vivo *and the IL-1β-induced production of NO and PGE_2 _in chondrocytes *in vitro*. Plasma samples collected before and 2 hr after supplementation with PFE were tested. Plasma samples collected after oral ingestion of PFE were found to inhibit the IL-1β-induced PGE_2 _and NO production in chondrocytes. These same plasma samples also inhibited both COX-1 and COX-2 enzyme activity *ex vivo *but the effect was more pronounced on the enzyme activity of COX-2 enzyme. Taken together these results provide additional evidence of the bioavailability and bioactivity of compounds present in pomegranate fruit after oral ingestion. Furthermore, these studies suggest that PFE-derived bioavailable compounds may exert an anti-inflammatory effect by inhibiting the inflammatory cytokine-induced production of PGE_2 _and NO *in vivo*.

## Background

Pomegranate has been used for centuries to confer health benefits in a number of inflammatory diseases. Based on its usage in Ayurvedic and Unani medicine, dietary supplements containing pomegranate extract are becoming popular in the Western world for the treatment and prevention of arthritis and other inflammatory diseases. More recently standardized extracts of pomegranate fruit (PFE) have been shown to possess anti-inflammatory and cartilage sparing effects *in vitro *[1]. Published studies have shown that constituents of PFE inhibit the proliferation of human cancer cells and also modulate inflammatory subcellular signaling pathways and apoptosis when directly added to the culture medium [2-6]. PFE has also been shown to significantly reduce the growth of prostate tumors and the levels of prostate-specific antigen (PSA) in nude mice implanted with prostate cancer cells [7]. Several groups have reported that consumption of pomegranate may have cholesterol lowering and cardiovascular and other chronic diseases preventing effects *in vivo *[8-11]. In these studies the major effect of the pomegranate extract consumption was the reduction of oxidative stress, inhibition of p38-mitogen-activated protein kinase (p38-MAPK) pathway and inhibition of the activation of transcription factor NF-κB. Activation of p38-MAPK and NF-κB is intimately associated with the increased gene expression of TNF-α, IL-1β, MCP1, iNOS and COX-2-agents that are critical mediators of inflammation and the pathogenesis of inflammatory and degenerative joint diseases [12,13]. These and other published studies [[14], reviewed in [15,16]] thus demonstrate that PFE possesses strong antioxidant and anti-inflammatory properties and its consumption has the potential to prevent diseases in which redox imbalance and inflammatory stimuli plays a decisive role.

The major class of phytochemical present in pomegranate is the polyphenols and includes flavonoids, condensed tannins and hydrolysable tannins. Hydrolysable tannins are predominant polyphenols found in pomegranate juice and account for 92% of its antioxidant activity [14]. Pomegranate seeds are rich in sugars, polyunsaturated (n-3) fatty acids, vitamins, polysaccharides, polyphenols, and minerals and have high antioxidant activity. When crushed and dried, the seeds produce an oil with 80% punicic acid, the 18-carbon fatty acid, along with the isoflavone genistein, the phytoestrogen coumestrol, and the sex steroid estrone. The seed coat of the fruit contains delphinidin-3-glucoside, delphinidin-3,5-diglucoside, cyanidin-3-glucoside, cyanidin-3,5-diglucoside, pelargonidin-3-glucoside, and pelargonidin-3,5-diglucoside with delphinidin-3,5-diglucoside being the major anthocyanin in pomegranate juice [11]. Studies have also shown that the antioxidant capacity of pomegranate juice is three times that of the popular antioxidant-containing beverages such as red wine and green tea, presumably due to the presence of hydrolyzable tannins in the rind, along with anthocyanins and ellagic acid derivatives [14]. In a comparative analysis, anthocyanins from pomegranate fruit were also shown to possess higher antioxidant activity than vitamin-E (α-tocopherol), ascorbic acid and β-carotene [17]. Pomegranate extract has also been shown to protect from NSAID and ethanol-induced gastric ulceration [18]. Repeated administration of high doses of a hydroalcoholic extract of pomegranate whole fruit or its constituent ellagitannin punicalagin were non toxic in the dosages commonly employed in traditional medicine systems [19,20].

Flavonoid rich fractions of pomegranate fruit extract have also been shown to exert antiperoxidative effect as their administration significantly decreased the concentrations of malondialdehyde, hydroperoxides and enhanced the activities of catalase, superoxide dismutase, glutathione peroxidase and glutathione reductase in the liver [21,22]. Anthocyanins were shown to be effective inhibitors of lipid peroxidation, the production of nitric oxide (NO) and inducible nitric oxide synthase (iNOS) activity in different model systems [22-24]. After consumption, anthocyanins are efficiently absorbed as glycosides from the stomach and are rapidly excreted into bile as intact and metabolized forms [25,26]. Plasma concentration of 30 μg/ml of punicalagin and 213 ng/ml of ellagic acid after oral administration in rats has been reported [27]. In humans it has been shown that ellagic acid is rapidly absorbed and plasma concentrations of 31.9 ng/ml were detected within one hour of oral consumption of pomegranate juice [28]. Cyclooxygenase (COX), an enzyme involved in the mediation of inflammatory process, catalyzes the rate-limiting step in the synthesis of prostaglandins from arachidonic acid [29,30]. Of its two isoforms, COX-1 is constitutively expressed in most tissues and appears to be responsible for maintaining normal physiological functions whereas COX-2 has been shown to be involved in cutaneous inflammation, cell proliferation, and skin tumor promotion [31]. These data suggest that inhibition of COX-2 activity is important for alleviating inflammation. Other studies have shown that Prodelphinidins isolated from *Ribes nigrum *inhibit cyclooxygenase-2 (COX-2) and lipoxygenase activity and production of prostaglandins E_2 _(PGE_2_) *in vitro*, suggesting that the primary effect of delphinidins (also present in pomegranate fruit) may be against inflammatory responses [32]. More recently it has been shown that pomegranate extract exerted a powerful influence in inhibiting the expression of inflammatory cytokines IL-1β and IL-6 in adjunctive periodontal therapy [33]. Other *in vitro *studies have shown that the bioactivity of total pomegranate extract was superior to its purified individual polyphenols illustrating the multifactorial effects and chemical synergy of the action of multiple compounds present therein [2].

While evidence from *in vitro *studies does not prove *in vivo *biological activity, these do provide a rationale and support for the use of pomegranate fruit or its extract to suppress inflammation *in vivo*. However, it is also important to point out that there are issues that deserve an explanation and require caution in interpreting the data obtained from *in vitro *studies. One question often raised is whether the concentration of a plant or fruit extract constituent compound that has been used in *in vitro *experiments would be realistic or achievable *in vivo*. In majority of the cases this has to be denied because constituents of plant or fruit extracts are typically not completely bioavailable and only certain constituents can be expected to be absorbed and become bioavailable via the hepatic portal system [34]. Another issue to be considered is that the bioeffective compounds do not necessarily need to be present in the original extract, but might be formed *in vivo *due to intestinal bacterial and/or hepatic metabolism [34]. This is supported by recent studies demonstrating that after ingestion of pomegranate juice by human volunteers ellagic acid metabolites which were not present in the juice consumed such as dimethylellagic acid glucuronide were detected in plasma and urine while Urolithins-formed by intestinal bacteria-were detected in the urine samples [35].

Pomegranate fruits are popularly consumed throughout the world and fruit and flower extracts are widely used for the treatment of inflammatory diseases in the traditional medicine systems of Asia and Europe. In this study using rabbits we determined whether after oral ingestion of a standardized preparation of pomegranate fruit extract (PFE), blood plasma samples contained PFE-derived metabolites/constituents by HPLC-DAD analysis. To test whether these same plasma samples exert anti-inflammatory effects, we determined whether the presence of these plasma samples in the assay mixture or culture medium can (a) inhibit the enzymatic activity of purified cyclooxygenases *ex vivo; *and (b) inhibit IL-1β-induced production of nitric oxide (NO) and PGE_2 _by rabbit articular cartilage chondrocytes *in vitro*.

## Methods

### Preparation of pomegranate fruit extract (PFE)

Pomegranate fruit (POMWonderful) was procured from the market and the extract was prepared essentially as previously described [1]. The filtrate was condensed and freeze-dried and stored at -20°C prior to use. For use required concentration of the freeze dried preparation was dissolved in sterile water.

### Total phenolics

The total phenolics were determined by the Folin-Ciocalteau method as previously described [36]. Briefly, 50 mg of the dried powder was extracted with 100 ml of acidified methanol:water (60:40 v/v, 0.3% HCl) and filtered. Filtrate was mixed with equal amounts of the Folin-Ciocalteau reagent (Sigma) and 2.0 ml of sodium bicarbonate was added and mixed thoroughly. After 2 h, absorbance was measured at 725 nm and the values were derived from a standard curve prepared using Tannic acid (0 – 1.0 mg/ml in acidified methanol:water). Values were expressed as mg/gm Tannic acid equivalents (mg/gm of TAE).

### Rabbits

For these studies we used 6 New Zealand white rabbits (male, 1 yr old, Average weight 3.7 Kg). Rabbits were acclimatized for one week and were then divided into 2 groups: (1) Experimental (4 rabbits); and (2) Control (2 rabbits). Rabbits in both the groups were food starved overnight and the next morning experimental rabbits were given 10 ml of PFE (34 mg/Kg) by gavage. Based on the phenolics content of PFE this dose was equivalent to 175 ml of pomegranate juice. The control rabbits were given just 10 ml of water the same way. Blood (10 ml) was collected prior to supplementation with PFE (Control plasma) and at 2 h post supplementation with PFE (Experimental plasma) in EDTA tubes (Becton Dickinson) and plasma was separated by standard methods and stored at -80°C prior to use.

### Extraction of anthocyanins from blood and HPLC analysis

The EDTA blood samples were centrifuged at 500 *g *for 10 min at 4°C, and the plasma was quickly removed. A 0.5 mL aliquot of plasma was acidified with acetic acid (10 mM) to prevent degradation of polyphenols related metabolites and was stored at -70°C until the analyses. For analysis by HPLC, 1 ml of acidified plasma was mixed with MeOH:0.2 M HCl (1:1, v:v), vortexed for one min and centrifuged at 14,000 g for 2 min at 4°C. The supernatant was filtered through a 0.45 μm filter and 10 μl of the filtrate was directly analyzed by HPLC-DAD using Agilent 1100 system on a reversed-phase C 18 column (Eclipse XDB 150 × 4.6 mm; particle size 5 μM). Solvent (A) was 0.1% (v/v) TFA/Water and solvent (B) was 0.1% TFA/Acetonitrile and a flow rate of 1 ml/min was maintained (initial 3% B, then 0–2 min 3% B; 2–32 min 3% – 60% B; 32 – 37 min 60% B; 37 – 38 min 60% to 3% B). Ellagic acid standard (Chromadex) was dissolved in DMSO and was found to elute at 24.6 min using the above described parameters.

### Preparation of chondrocytes and treatment

Rabbit chondrocytes were prepared from the articular cartilage by enzymatic digestion as previously described for human chondrocytes [1,37]. Chondrocytes were plated (1 × 10^6^/ml) in 48 well culture plates (Becton-Dickinson, Franklin Lakes, NJ) in complete DMEM with 10% foetal calf serum and allowed to grow for 72 h at 37°C and 5% CO_2 _in a tissue culture incubator. Chondrocytes (>80% confluent) were serum-starved overnight and then pre-treated with either control or experimental rabbit blood plasma for 2 hrs and then stimulated with IL-1β (5 ng/ml) for 24 hrs. Chondrocytes cultured without IL-1β served as controls in all of the experiments. Cell viability before plating was monitored by the MTT assay (Cell Viability and Proliferation Assay) according to the instructions of the manufacturer (R&D Systems). In some cases, viability of chondrocytes after exposure to PFE and IL-1β was determined by Trypan blue exclusion assay.

### Determination of COX activity by EIA

The COX-1 and COX-2 inhibitory assay was carried out using a COX Inhibitor Screening Assay Kit (Cayman Chemicals, Ann Arbor, MI) according to the instructions provided with the kit. Briefly, heme and COX enzymes were added to the tubes containing the kit supplied reaction buffer and the mixture was vortexed and mixed with either reaction buffer or an aliquot (20 μl) of plasma sample diluted 5 fold in the same buffer and incubated at 37°C for 10 min. Acetylsalicylic acid was used as positive control. Arachidonic acid solution was then added to the tubes to start the cyclooxygenase reaction and after incubation at 37°C for 2 min, 1M HCl was added to terminate the reaction. PGH_2 _formed was reduced to PGF_2α _with saturated stannous chloride solution. The COX activity was measured based on the amount of PGF_2α _detected by the enzyme immunoassay kit using a standard curve. The COX enzyme inhibitory activity of plasma samples obtained before the oral ingestion of PFE (Control) was compared to COX enzyme activity inhibition induced by plasma samples obtained 2 h after the oral ingestion of PFE (Experimental). For each measurement, control and experimental plasma samples obtained from the same rabbit were used. Values obtained were expressed a percent COX enzyme activity remaining relative to activity of the control enzyme (kit supplied) which was taken as 100% activity when the assay was performed in the absence of inhibitors.

### Determination of nitric oxide

The nitrite concentration in the chondrocytes culture medium was measured by the Griess reaction as an indicator of NO production. Briefly, 100 μl of culture supernatant was mixed with 900 μl of Griess reagent (1% sulphanilamide in 5% phosphoric acid and 0.1% naphthylethylenediamine dihydrochloride in water) and incubated for 15 min at room temperature. Absorbance of the mixture at 540 nm was determined using λ 25 Spectrophotometer (Perkin-Elmers, CT) and the concentration was derived using a standard curve prepared with sodium nitrite.

### Measurement of PGE_2 _production

Levels of PGE_2 _in the chondrocytes culture supernatant were quantified using a commercially available kit (R & D Systems, Cat# KGE004) according to the instructions provided with the kit.

### Statistical analysis

Experiments were repeated and each assay was performed in triplicate. Data was analyzed using the InStat 3.0 (GraphPad) software package (unpaired two tailed t-test with Welch correction) and *P *< 0.05 was considered significant. Values shown are Mean ± SE of Mean unless stated otherwise.

## Results

### PFE-derived metabolites in the blood

**Figure 1:**
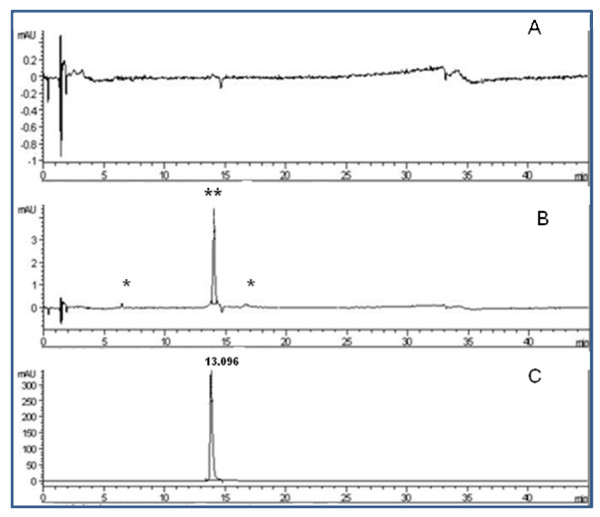
**Pomegranate constituents and metabolites are present in blood plasma after oral ingestion of an anthocyanin and hydrolysable tannin rich extract**. Representative HPLC chromatograms of plasma samples collected from rabbits before (A) and 2 h after consumption of PFE (B). Peak with double asterisk in B has the elution profile identical to that of purified ellagic acid standard shown in C. Peaks with single asterisk in C were detected only in plasma samples obtained after the oral ingestion of PFE but not in control plasma samples (blood drawn before feeding PFE).

The known antioxidant and antiatherosclerotic properties of pomegranate are mainly attributed to the high content of polyphenols, including hydrolysable tannins and ellagitannins (ET), present in the pomegranate fruit [14]. The extract was found to contain 107.5 ± 3 mg/g total polyphenolics expressed as tannic acid equivalents (TAE, mg/g of TAE). The HPLC chromatogram of the PFE used in this study showed the presence of several polyphenols including ellagic acid (EA) (at t_R _24.6 min, results not shown). For the HPLC analyses ellagic acid was used as a marker since EA has been shown to become bioavailable after oral consumption of pomegranate juice and the presence of EA in blood and urine has been suggested as a reliable marker for assessing compliance in studies involving the consumption of pomegranate fruit [35]. Control plasma samples showed no peak corresponding to EA on HPLC chromatogram (Figure 1A) while a peak corresponding to EA was detected in the plasma samples obtained 2 h after the ingestion of PFE from the same animal (Figure 1C and results not shown). Additional peaks detected in the experimental plasma samples at t_R _27.9, t_R _34.1, t_R _34.7 and t_R _36.8 (Figure 1C &1D) were also not detected in the control blood samples (Figure 1A &1B) and therefore are likely to be PFE-derived. These results confirm the previous findings [26-28,35] and demonstrate that PFE constituents and PFE-derived metabolites become bioavailable after oral ingestion.

### Inhibition of COX activity

**Figure 2:**
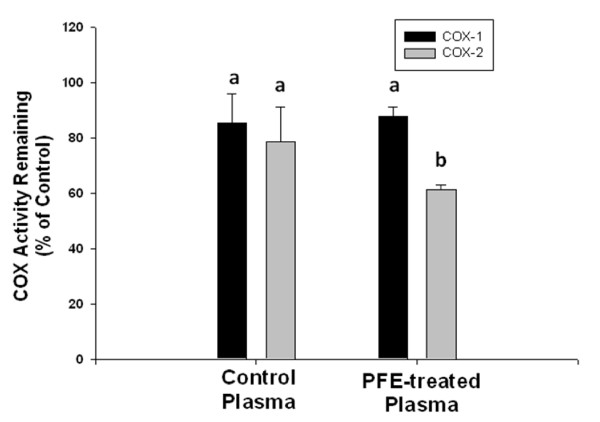
**Suppression of COX 1 and COX 2 enzyme activity by plasma of rabbits 2 h after oral administration of PFE**. Enzyme activity of COX 2 but not of COX 1 was inhibited significantly (*P *< 0.05) compared to control by plasma samples obtained 2 h after the oral ingestion of PFE (PFE-treated plasma). Suppression of COX 1 and COX 2 enzyme activity by control plasma samples did not reach statistical significance compared to purified enzymes provided in the kit (*P *> 0.05). Acetylsalicylic acid was used as positive control for inhibition of COX 1 and COX 2 enzyme activity and showed 100% inhibition at the concentrations used. Data shown is Mean ± SE derived from 4 experimental and 2 control plasma samples, each run in duplicate and differ without a common letter (*P *< 0.05)

After ingestion of a concentrated dose of PFE, the incubation of plasma samples with purified COX-1 and COX-2 enzymes showed a direct inhibitory effect on the enzyme activity (Figure 2). In the assay procedure, plasma was diluted 10 fold before the COX reaction was started. Incubation with plasma samples obtained before the oral ingestion of PFE suppressed the COX-1 activity by 14.85 ± 2.41% while incubation with blood samples obtained after supplementation with PFE suppressed the COX-1 activity by 21.47 ± 3.64%. This inhibition of COX-1 enzyme activity when post-supplementation plasma was added directly in the assay system was statistically significant when compared to the activity level in controls (*P *< 0.05). In contrast, incubation of COX-2 enzyme with pre-supplementation plasma inhibited the enzyme activity by 12.27 ± 4.79% (*P *> 0.05 compared to control) but incubation with post supplementation plasma inhibited the COX-2 activity by 38.8 ± 9.59% and this inhibition of COX 2 enzyme activity was statistically highly significant (*P *< 0.05). The mean PGF_2α _concentrations detected after incubation of COX-1 enzyme with arachidonic acid in the presence of pre-supplementation plasma samples were 254.33 ± 4.5 ng/ml and 247.66 ± 14.97 ng/ml after incubation of the enzyme with its substrate in the presence of post-supplementation plasma. When COX-2 enzyme was incubated with pre-supplementation plasma, the mean PGF_2α _concentration detected was 592.00 ± 91.00 ng/ml. In sharp contrast concentrations of the PGF_2α _were dramatically reduced to 199.33 ± 32.39 ng/ml when COX-2 enzyme and its substrate were incubated with the post-supplementation plasma samples. These data clearly indicate that the enzyme activity of COX-2 was significantly influenced by PFE constituents or metabolites that become bioavailable in the plasma after oral ingestion. The COX-2/COX-1 ratio of inhibitory activity of the different plasma samples was determined as previously described [38] and was less than 1 for all of the samples with the mean ratio being 0.80 ± 0.071 indicating selective inhibition of COX-2.

### Inhibition of IL-1β-induced PGE_2 _production in chondrocytes

**Figure 3:**
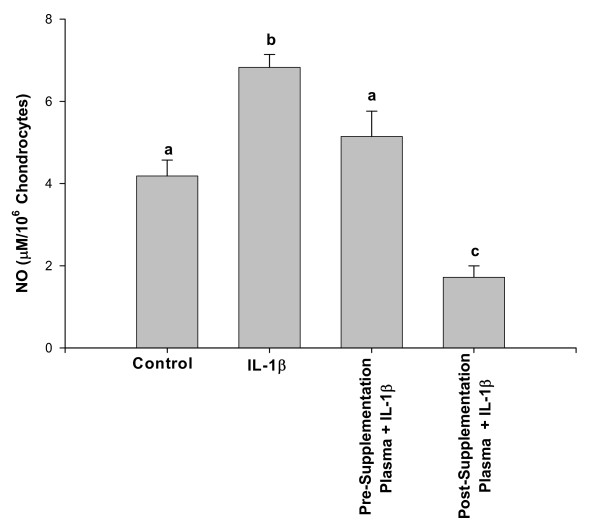
**Effect of Plasma samples obtained before and 2 h after oral ingestion of PFE on IL-1β-induced NO production in rabbit chondrocytes**. Confluent chondrocytes were serum starved and then treated with 200 μl of control or experimental plasma samples for 1 hr, stimulated with human IL-1β for 24 hrs. At the end of incubation, 100 μl of the medium was removed for measuring nitrite production by Griess reaction. Control values were obtained in the absence of plasma or IL-1β. Data were derived from two independent experiments, each run in triplicate, and expressed as Mean ± SE. Values without a common letter differ (*P *< 0.05 a vs b; *P *< 0.005, a vs c; b vs c).

As our studies showed that plasma containing bioavailable PFE constituents and PFE-derived metabolites was a potent inhibitor of COX activity *ex vivo*, we determined its effect on IL-1β-induced production of PGE_2 _in articular cartilage chondrocytes *in vitro*. Levels of PGE_2 _in the culture medium were estimated using an ELISA based assay. As shown in Figure 3, control chondrocytes and chondrocytes treated with either plasma samples alone produced only low levels of PGE_2_. Stimulation of chondrocytes with IL-1β produced a dramatic rise in the level of PGE_2 _in the culture medium indicating enhanced eicosanoid generating enzyme activity in chondrocytes. Interestingly, chondrocytes stimulated with IL-1β in the presence of control plasma showed no inhibition of PGE_2 _production while significantly low levels of PGE_2 _were detected in chondrocyte cultures stimulated with IL-1β in the presence of experimental plasma samples (Figure 3, *P *< 0.005).

### Inhibition of IL-1β-induced NO production in chondrocytes

**Figure 4:**
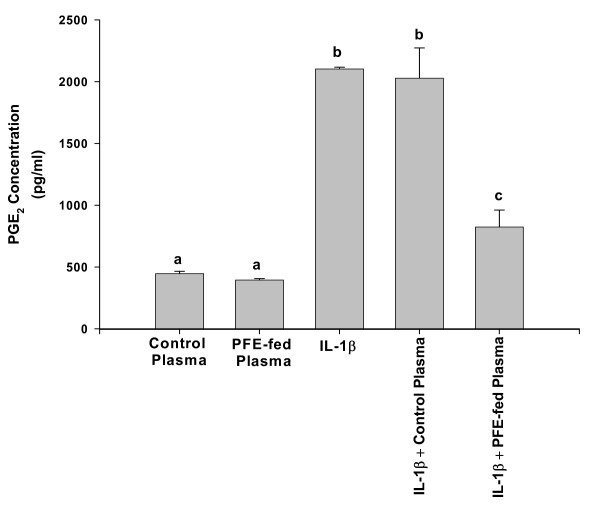
**Plasma samples obtained 2 h after oral ingestion of PFE inhibited IL-1β-induced PGE_2 _production by chondrocytes**. Confluent chondrocytes were serum starved and then treated as described for Figure 3 above. The amount of PGE_2 _produced in the medium was measured as described in Materials and Methods. Data were derived from two independent experiments, each run in duplicate. Values shown are Mean ± SE and differ without a common letter (*P *< 0.005).

Previous studies have shown that pomegranate extract was an effective inhibitor of NO in different systems [10,39,40]. However, whether blood plasma containing bioavailable pomegranate-derived metabolites also suppress cytokine-induced NO production was not investigated in these or other published studies. In the present study, effect of bioavailable pomegranate-derived metabolites on IL-1β-induced NO production in rabbit chondrocytes was investigated. Accumulation of nitrite in the culture medium was determined by the Griess reaction and was used as an index for NO synthesis by chondrocytes. As shown in Figure 4, unstimulated rabbit chondrocytes produced background levels of NO in the culture medium. When chondrocytes were stimulated with IL-1β, nitrite concentration in the medium increased significantly, about 2.5 fold, (*P *< 0.05). When chondrocytes were pre-treated with pre-supplementation plasma and then stimulated with IL-1β for 24 h, the production of NO was reduced approximately by 25% (5.14 μM). In contrast, a dramatic and highly significant reduction in nitrite accumulation was noticed in culture medium when chondrocytes were pre-treated with plasma obtained 2 h after the oral ingestion of PFE and then stimulated with IL-1β for 24 h (0.90 μM, *P *< 0.005). When cell viability was checked using the Trypan Blue exclusion assay, results indicated that incubation of chondrocytes with pre- or post-supplementation plasma did not decrease the viability of chondrocytes (results not shown). This indicated that the inhibition of IL-1β-induced NO and PGE_2 _production reported in this study was not a cytotoxic effect of pomegranate-derived metabolites present in the plasma.

## Discussion

The health promoting effects of plant constituents and extracts are being increasingly studied and their consumption is on the rise in the western world [41-43]. Although several studies have reported the effectiveness of different herbal preparations or fruit extracts for the treatment and/or prevention of chronic diseases [reviewed in [43]], bioavailability of the active principle(s), which could also be metabolically derived, must be evaluated in order to provide a valid explanation for the observed or reported bioefficacy. This is more so as the plant or fruit extracts are a complex mixture of various constituents and in most of the instances it is not clear whether a single compound or a mixture of compounds is responsible for the observed or reported effect [34]. However, evidence is accumulating that often related compounds present in a herb or fruit extract augment each other's biological effect. For example, it has been reported that ellagic acid and quercetin (both are also present in pomegranate) together exert a more pronounced inhibitory effect against cancer cell growth than either compound alone [2].

Arthritis (Osteoarthritis and rheumatoid arthritis) is one of the most prevalent and disabling chronic diseases of the diarthrodial joints and mostly affect the elderly. Cure for arthritis is still elusive and the management of the disease is largely palliative focusing on the alleviation of symptoms. Current recommendations for the management of arthritis include a combination of non-pharmacological interventions (weight loss, education programs, exercise, etc) and pharmacological treatments (paracetamol, nonsteroidal antiinflammatory drugs-NSAIDs, biologics, etc). Among these pharmacological treatments, NSAIDs, despite serious adverse effects associated with their long-term use, remain among the most widely prescribed drugs for relieving the pain of arthritis [44]. This highlights a need for safe and effective alternative treatments while the absence of any cure reinforces the importance of prevention. The prevention and alternative treatments could come from nutrition. It is now becoming increasingly clear that, beyond meeting basic nutritional needs, consumption of certain foods may play a beneficial role in the prevention of some chronic diseases [45]. Arthritis being a chronic disease is the perfect paradigm of a pathology whose prevention and/or treatment could potentially be addressed by nutrition. This is because, in most cases, a biologically active dietary constituent has only limited effects on its target and relevant and significant differences are only reached over time through a cumulative effect where daily benefits add up day after day [46]. However, bioavailability of plant, fruit or herb constituents or metabolites after consumption and their bioactivity must be studied before making a recommendation. In the present study we used an experimental approach in which absorption and metabolism of constituents of the popular and exotic fruit pomegranate were taken into consideration with a view to gain an insight into the basis of the reported *in vivo *anti-inflammatory and chemopreventive effects of its consumption on human health [reviewed in [15,16]]. Our data show that PFE constituents, with EA being one of them, become bioavailable 2 h after oral ingestion of a modest amount of concentrated pomegranate extract and that a value of 247 ng EA/ml of plasma was detected. This is very similar to the values detected in rats [27] but in humans levels of EA detected in the plasma after consumption of pomegranate juice concentrate were low [28], at least at the time points analyzed. This difference may be due to the differences in the metabolism or clearance rate between humans and rabbits. Additionally, EA is poorly soluble in water and is reported to accumulate in the human intestinal epithelial cells [47]. These factors could also contribute to its lower levels reported in human plasma. We also show here for the first time that after oral ingestion of PFE, constituents of PFE or their metabolites that become bioavailable in plasma significantly inhibited the activity of COX-1 and COX-2 enzymes in a direct enzyme inhibition assay with the inhibitory effect being targeted more towards COX-2. These results suggest that these constituents of PFE or compounds derived from them may prove to be more potent but non-toxic or less toxic inhibitors of COX-2. Further research is needed before reaching a conclusion in this regard. We also show that bioavailable constituents or metabolites of PFE present in the plasma were biologically active against inflammatory mediators as they also inhibited the inflammatory stimuli-induced production of NO and PGE_2 _in chondrocytes. These results are therefore relevant for strategies designed to prevent cartilage degradation in arthritic joints and support further studies in animal models.

There are large numbers of phytochemicals consumed in our diet and among them polyphenols constitute the largest group. Although direct inhibitory effect of plant extracts or components on COX enzyme activity have been reported by several investigators [47-55] but inhibition of COX enzyme activity by polyphenols that become bioavailable after consumption of pomegranate fruit or extract has not been reported. As we focus on the prevention and treatment of arthritis by natural products, in a previous report we showed that pomegranate extract was effective in suppressing the IL-1β-induced human cartilage matrix proteoglycan release *in vitro *[1]. In this report we have addressed the *in vivo *efficacy of pomegranate constituents and/or their metabolites that become bioavailable after oral ingestion PFE. It is also important to point out that the polyphenolic content of the PFE powder (34 mg/Kg) employed in this study was equivalent to the polyphenolic content of 175 ml of pomegranate juice indicating that this is feasible in terms of human nutrition. Inhibition of COX activity by constituents and/or metabolites that became bioavailable via systemic circulation provide the first direct evidence of pomegranate extract-derived active principles in the plasma that significantly inhibited the COX-2 activity (*P *< 0.05). After the oral ingestion of a single dose of PFE the inhibition of COX-1 and COX-2 induced by rabbit plasma samples indicated a COX-2/COX-1 ratio of 0.8 which is suggestive of selective inhibition of COX-2 [38]. Selective COX-2 inhibition with COX-2/COX-1 ratios below 1 was previously reported for resveratrol and its analogues [56] but selective inhibition of COX-2 by bioavailable constituents or metabolites of a fruit or plant extract has not been shown. In another study, bioavailability and COX inhibitory activity of Pycnogenol constituents or their metabolites in human serum was studied, but in this study the effect was not found to be COX-2 selective as the COX-2/COX-1 activity ratio was greater than 1 [34]. In a chronic gastric ulcer model, consumption of sangre de grado extract selectively suppressed the COX-2 mRNA expression in the ulcer bed but the effect on COX activity was not studied [57]. Although COX-1 is constitutively expressed while COX-2 is induced in an inflammatory response, use of plant extracts or isolated polyphenols directly in *in vitro *assays to inhibit COX activity fails to address the question whether sufficiently high concentrations of these flavonoids could be achieved *in vivo *to exert the same effect [34]. Our results address this question and also provide support to the reported use of pomegranate extract for the treatment of inflammatory bowel diseases or gastric ulcers by the practitioners of Ayurveda and Unani systems of medicine [58].

Results of the present study also highlight the effectiveness of bioavailable pomegranate fruit constituents and/or metabolites present in the blood plasma to inhibit the IL-1β-induced NO production in articular cartilage chondrocytes. Biological activities of polyphenols present in popular medicinal plants and herbs have been studied extensively including inhibition of inflammatory stimuli-induced responses in different cell and tissue types [reviewed in [14]]. NO plays a pivotal role as second messenger and an effecter molecule in a variety of tissues. NO also have been defined as an important molecule in inflammation and to the pathogenesis of osteoarthritis (OA) as excessive production of NO induced by inflammatory cytokines in chondrocytes and other cell types in arthritic joints has been related to the induction of apoptosis in chondrocytes [59]. Therefore, compounds that inhibit excessive NO production may have beneficial therapeutic effects in arthritis by blocking cartilage degradation. However, this needs to be evaluated first in an animal model followed by controlled clinical trials.

## Conclusion

These studies provide evidence to show that bioavailable constituents and/or metabolites of PFE exert an anti-inflammatory effect by inhibiting the activity of eicosanoid generating enzymes and the production of NO. This further suggests that consumption of PFE may be of value in inhibiting inflammatory stimuli-induced cartilage breakdown and production of inflammatory mediators in arthritis.

## Competing interests

The authors declare that they have no competing interests.

## Authors' contributions

MS carried out the experimental work, collected and interpreted the data, KG carried out the experimental work, collected and interpreted the data, ZR carried out the experimental work, collected and interpreted the data, KAK participated in literature search and drafting of the manuscript, TMH conceived of the study, its design, coordination and drafting the manuscript.

All authors have read and approved the final manuscript.
